# Ultrasound-Guided Genicular Nerve Blocks for Anterior Cruciate Ligament Reconstruction Surgery in an Outpatient Setting: A Case Series

**DOI:** 10.7759/cureus.44550

**Published:** 2023-09-01

**Authors:** Vendhan Ramanujam, Stephen DiMaria, Vivek Varma

**Affiliations:** 1 Anesthesiology, Rhode Island Hospital/The Warren Alpert Medical School of Brown University, Providence, USA

**Keywords:** outpatients, anterior cruciate ligament reconstruction, analgesia, ultrasonography, genicular nerve blocks

## Abstract

Arthroscopic knee anterior cruciate ligament (ACL) reconstruction is commonly performed as an outpatient surgery, where adequate pain control and early ambulation play key roles in recovery and discharge. Peripheral nerve blocks aid in this purpose. Blockade of the genicular nerves, the articular branches of the knee, has recently become popular for knee surgery. We report on four patients who underwent ACL reconstruction with ultrasound-guided genicular nerve blocks (GNBs) under general anesthesia. The blocks were reliably performed without any complications, and the patients experienced good pain control, reduced opioid intake, and timely discharge following the surgery. These findings necessitate future investigations into the use of GNBs in ACL reconstruction.

## Introduction

Knee arthroscopy procedures, such as anterior cruciate ligament (ACL) reconstruction, are commonly performed knee surgeries where pain control and early ambulation play important roles in recovery and discharge. The introduction of regional anesthesia techniques for knee surgery has substantially aided this purpose by reducing pain and opioid consumption. Initially, femoral and sciatic nerve blocks were commonly used but were associated with motor blockade, an increased risk of falls, delayed ambulation, and delayed discharge. These motor blocks have been replaced by sensory blocks, such as adductor canal block (ACB) and Infiltration between the Popliteal Artery and Capsule of the Knee (IPACK) block, which provide analgesia without affecting the motor strength of the lower extremity. A combination of both these blocks has demonstrated positive outcomes in knee arthroscopy surgery [1, 2, 3]. However, there is interest in investigating finer and more selective blockade techniques. This is because the knee joint has a complex neural innervation, formed by several nerves from the lumbar-sacral plexus-i.e., femoral, obturator, and sciatic-that neither ACB nor IPACK blocks can fully cover. All the above nerves and their branches combine to form terminal articular branches known as the genicular nerves, which enter the knee joint from different directions (superolateral, superomedial, inferolateral, inferomedial) [4]. Hence, blockade of these nerves can provide analgesia to the knee. Genicular nerve blocks (GNBs) have recently gained popularity and have shown promising results in total knee arthroplasty [5, 6]. Although these results could potentially translate to knee arthroscopy surgery, there is a paucity of studies that specifically investigate the use of GNBs for less invasive surgeries like ACL reconstruction, which is usually performed in an outpatient setting. We present a case series of retrospectively reviewed patients who received ultrasound-guided GNBs for arthroscopic ACL reconstruction surgery.

## Case presentation

As per our institution's policy, this case report series is exempt from institutional review board (IRB) review requirements since it is devoid of patient-identifiable information. The IRB waived the requirement for patient consent and/or provided a waiver for the patient authorization of the release of Protected Health Information (PHI) (written Health Insurance Portability and Accountability Act (HIPAA) authorization). Consent from the patients was obtained for the submission of this report. This manuscript adheres to the applicable Case Reports (CARE) guidelines. The case series includes four patients who underwent primary total knee arthroscopy for ACL repair with allografts at a tertiary academic medical center. All of them were healthy, had a normal body mass index, were classified as American Society of Anesthesiologists I or II, had no significant medical history, and showed normal physical examination and laboratory values. Their characteristics are provided in Table 1.

**Table 1:** Patient Characteristics BMI: body mass index; ASA: American Society of Anesthesiologists classification.

Patient	Age (years)	Sex	BMI (kg/m^2^)	ASA
1	38	Female	28	I
2	24	Male	26.8	I
3	18	Female	24.4	I
4	24	Female	26	II

**Figure 1:**
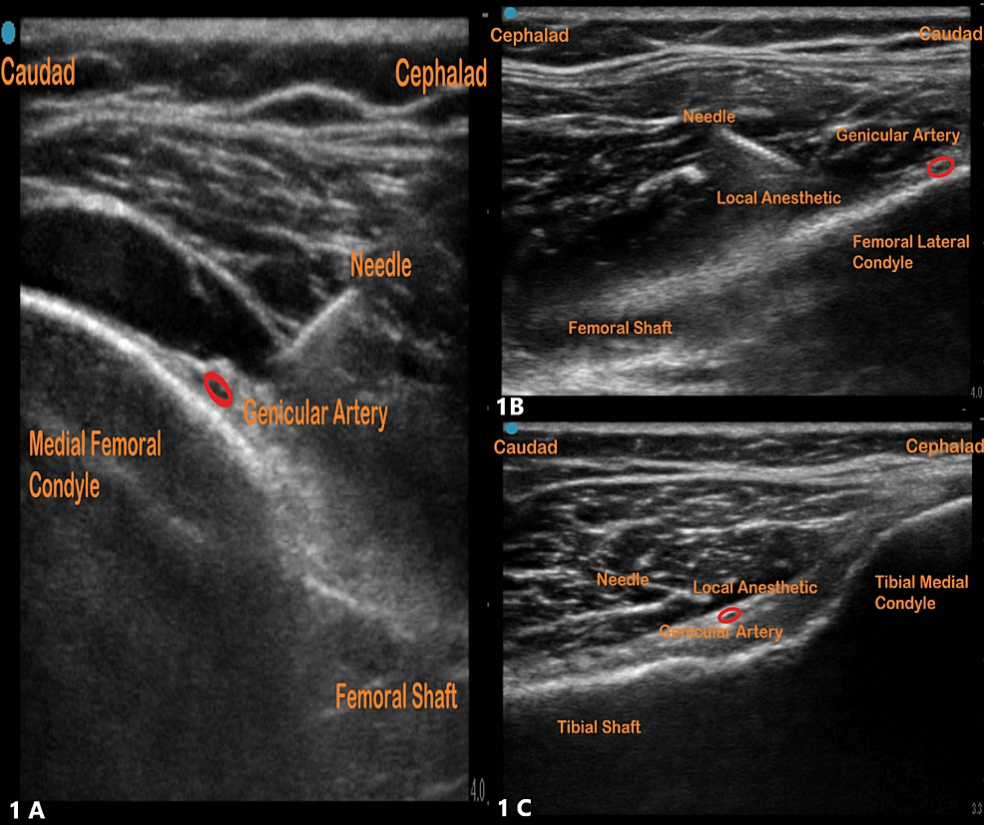
Ultrasound images of genicular nerve blocks. 1A: Superomedial genicular nerve block. 1B: Superolateral genicular nerve block. 1C: Inferomedial genicular nerve block.

General anesthesia, along with GNBs (superolateral, superomedial, inferomedial), was used as peripheral nerve blocks to aid with analgesia. The inferolateral GNB was avoided to prevent unintended motor blockade and weakness. The blocks were performed either before (one patient) or after induction (three patients), as per patient preference. A 22-gauge x 80 mm echogenic needle [PAJUNK®, SonoPlex® Stim II with Facet Tip] was used, along with ultrasound guidance [high-frequency (6-13 MHz) linear transducer; Fujifilm SonoSite, Inc., Bothell, WA, USA], standard monitoring, as-needed premedication (intravenous midazolam 2-4 mg), and sterile techniques. It took less than 15 minutes to place the nerve blocks for each patient. All procedures were performed by resident trainees under the supervision of an experienced regional anesthesiologist. There were no contraindications or complications associated with any of the blocks. GNBs were performed around the knee and along the long axis of the medial and lateral femur and medial tibia. In-plane needle insertion was directed toward the bone adjacent to the genicular arteries, and injectate was applied over the bone surface, resulting in elevation of the overlying soft tissue [5]. A 5 ml dose of 0.25% bupivacaine was administered for each GNB. The ultrasound images representing the GNBs are shown in Figure 1.

All the patients were induced with propofol (2 mg/kg) and received general anesthesia, in which a laryngeal mask airway was used for ventilation and sevoflurane (1.8%) maintained a minimum alveolar concentration of 1. Dexamethasone (4 mg) and ondansetron (4 mg) were administered for prophylaxis against postoperative nausea and vomiting. No intraoperative muscle relaxants or non-opioid pain medications were used. All patients received 950 milligrams of acetaminophen preoperatively. Intraoperative and postoperative opioids (intravenous hydromorphone and oral oxycodone) were administered as needed by the primary anesthesia team. A thigh tourniquet was used prior to incision in all the patients, and none received surgeon-administered local anesthetics. Nerve block-related data for each patient is presented in Table 2. The duration of surgery (from start to end) ranged between 79 and 101 minutes, and the duration of thigh tourniquet use ranged between 67 and 90 minutes. All patients reported a preoperative pain score of zero as per the numeric rating scale (NRS), which measures pain severity at that moment in time using a zero-to-ten scale, with zero indicating 'no pain' and ten indicating 'the worst pain imaginable.' The postoperative mean NRS pain scores ranged between zero and four, and postoperative opioid consumption ranged between 2.5 and 7.5 milligrams in morphine milligram equivalents (MME). MME was calculated by obtaining the dosage of each opioid administered and multiplying it by its respective Centers for Disease Control and Prevention conversion factor [7]. All patients were discharged home after recovery without any significant events and reported satisfactory pain control during the follow-up the day after the surgery.

**Table 2:** Nerve Block Related Data NRS: numeric rating scale; MME: morphine milligram equivalents.

Patient	Surgery duration (minutes)	Torniquet time (minutes)	Preoperative NRS pain score	Postoperative mean NRS pain score	Perioperative hydromorphone (milligrams)	Perioperative oxycodone (milligrams)	Perioperative MME (milligrams)	Recovery duration (minutes)
1	88	76	0	2	0.5	0	5	98
2	80	69	0	4	0.25	0	2.5	101
3	101	90	0	3	0	5	7.5	92
4	79	67	0	0	0.25	0	2.5	88

## Discussion

The nerve supply to the knee joint is complex, originating from the lumbar-sacral plexus. Studies have shown that branches from the plexus, such as the saphenous, obturator, and vastus (lateralis, medialis, intermedius), along with fibular nerves, innervate the anterior part of the knee. The fibular, tibial, and obturator nerves innervate the posterior part of the knee [4, 8-10]. These nerves form a group of terminal articulating nerves known as the genicular nerves. Tibial nerve branches give rise to the superomedial and inferomedial genicular nerves, while common peroneal nerve branches form the superolateral and inferolateral genicular nerves. Supra and infrapatellar genicular nerves are formed by the saphenous nerves. Obturator nerve contribution to these nerves is typically variable [9]. Given that the portal entries for arthroscopic ACL repair are through anteromedial and anterolateral directions, followed by femoral and tibial tunneling for graft insertion, blocking the genicular nerves can provide effective pain control during and after the surgery. This was observed in this report, where low pain scores and reduced opioid use were seen compared to studies evaluating other nerve blocks (femoral or ACB), local infiltration, and pharmacological agents used alongside general anesthesia for ACL reconstruction [11-13]. By avoiding blockade of the inferolateral genicular nerve, which receives an articular branch from the common peroneal nerve, unintended motor weakness was spared, and postoperative ambulation was unaffected [4]. With good pain control and early ambulation, recovery is enhanced, and timely discharge was possible following the GNBs where time to discharge was well within what has been previously reported as the average discharge time for ACL repair involving general anesthesia and other nerve blocks [13].

Although GNBs have traditionally been performed using fluoroscopy, recent knowledge about the precise locations of genicular nerves has made ultrasound-guided GNBs feasible [4, 8, 9]. Using the genicular arteries at the distal ends of the femur and tibia as landmarks, needle and injectate placement over these bones allows for local anesthetic spread and reliable blockade of the small genicular nerves that consistently come in contact with the bone close to the knee joint, even though their proximal or distal trajectories are variable [10]. Ultrasound-guided techniques have shown less pain, additional pathology detection, and avoidance of radiation exposure compared to blind and fluoroscopy-guided techniques. Additionally, these blocks can be performed quickly without the need for changing patient position, making ultrasound-guided GNBs ideal for ambulatory knee surgery.

This report has limitations. Being a case series, its findings are retrospective, not directly comparable to other blocks, and may be over-interpreted. The analgesic effect of ultrasound-guided GNBs for ACL reconstruction cannot be generalized or validated. The quality of recovery following the GNBs also cannot be determined.

## Conclusions

Ultrasound-guided GNBs for ACL reconstruction can be safely performed for pain control in an outpatient surgery setting. Excluding the inferolateral GNB can avoid motor weakness and assist in early ambulation. However, this is only a case series and future studies are needed to verify their efficacy and comparativeness with other peripheral nerve blocks that are currently in practice for ACL reconstruction.
